# The Value of the Galactomannan Test in Diagnosing COVID-19–Associated Pulmonary Aspergillosis: A Review 

**DOI:** 10.30699/ijp.2025.2044324.3369

**Published:** 2025-03-10

**Authors:** Mohammadreza Salehi, Jon Salmanton-García, Alireza Abdollahi, Maryam Albaji, Effat Davoudi-Monfared, Zeinab Siami, Saeed Mohammadi, Sadegh Khodavaisy, Pershang Nazemi

**Affiliations:** 1 Research Center for Antibiotic Stewardship & Antimicrobial Resistance, Department of Infectious Diseases, Imam Khomeini Hospital Complex, School of Medicine, Tehran University of Medical Sciences, Tehran, Iran; 2 University of Cologne, Faculty of Medicine, and University Hospital Cologne, Institute of Translational Research, Cologne Excellence Cluster on Cellular Stress Responses in Aging-Associated Diseases (CECAD), Cologne, Germany; 3 University of Cologne, Faculty of Medicine, University Hospital Cologne, Department I of Internal Medicine, Center for Integrated Oncology Aachen Bonn Cologne Duesseldorf (CIO ABCD) and Excellence Center for Medical Mycology (ECMM), Cologne, Germany; 4 German Center for Infection Research (DZIF), Partner Site Bonn-Cologne, Cologne, Germany; 5 Department of Pathology, School of Medicine, Imam Khomeini Hospital Complex, Tehran, Iran; 6 Department of Pulmonary Diseases, Sina Hospital, School of Medicine, Tehran University of Medical Sciences, Tehran, Iran; 7 Department of Clinical Pharmacy, Yas Hospital Complex, School of Pharmacy, Tehran University of Medical Sciences, Tehran, Iran; 8 Department of Infectious Diseases, School of Medicine, Ziaeian Hospital, Tehran University of Medical Sciences, Tehran, Iran; 9 Department of Epidemiology and Biostatistics, School of Public Health, Tehran University of Medical Sciences, Tehran, Iran; 10 Department of Medical Parasitology and Mycology, School of Public Health, Tehran University of Medical Sciences, Tehran, Iran; 11 Department of Infectious Diseases, Yas Hospital Complex, Tehran University of Medical Sciences, Tehran, Iran

**Keywords:** Galactomannan, Aspergillosis, SARS-CoV-2, COVID-19, Bronchoalveolar lavage, Fungi, Viral Diseases, Invasive fungal infections

## Abstract

COVID–19–associated pulmonary aspergillosis (CAPA) is a complication of COVID-19. Galactomannan (GM) is a non-invasive test used to diagnose invasive aspergillosis. We collected the existing studies on the diagnostic value of GM to determine a GM level for predicting CAPA. All articles on the value of GM in CAPA diagnosis published until November 2023 were reviewed. The main databases were searched using the following keywords: “aspergillus”, “aspergillosis”, “SARS-CoV-2”, “COVID”, “2019 ncovnCOV”, “novel coronavirus”, “COVID-19”, “galactomannan”, and “CAPA”. Studies with reported levels of serum or BAL GM were included. Patients were classified into two groups: non-confirmed and proven aspergillosis. Finally, the receiver operating characteristic (ROC) curve analysis was used to determine a GM level to predict the likelihood of CAPA. A total of 26 articles were selected, of which 239 patients were included. A count of 123 patients (50%) were in the non-confirmed group and 124 (50%) patients were proven. The median serum GM was 0.51 in the non-confirmed group and 0.47 in the proven group (p= 0.73). The level of GM in BAL fluid was 0.10 in the non-confirmed and 2.80 in the proven group, which was statistically different (p<0.001). With 81.3 % sensitivity and 79.5% specificity, the BAL GM cut-off was 1.01 ODI. The results showed that BAL GM ≥1.01 can be used to predict CAPA. Serum GM did not show any predictive value in diagnosing CAPA. However, BAL GM level can be a reliable diagnostic test in patients with CAPA.

## Introduction

Invasive fungal infections, including invasive pulmonary aspergillosis (IPA), primarily affect severely immunocompromised patients, such as leukemia patients with chemotherapy-induced neutropenia or transplant recipients (1, 2). However, over the past two decades, the clinical spectrum of IPA has expanded to include new categories of "at-risk" patients, such as those in intensive care units (ICU) without underlying immunosuppressive conditions (3, 4). The increased risk of IPA among patients who required ICU admission and mechanical ventilation was brought to attention during the H1N1 influenza pandemic of 2009-2010 (5, 6). Similarly, the COVID-19 pandemic raised concerns regarding the development of IPA in patients with severe respiratory symptoms due to the disturbance of immune homeostasis (7-11). As the pandemic continued, more cases of IPA were identified, and various treatment strategies were proposed. However, early diagnosis was challenging due to the novel characteristics of COVID-19 (such as lymphopenia), inherent challenges in diagnosing fungal infections, and resource constraints during the pandemic (12). The emergence of IPA due to diverse causes in ICU patients led to the establishment of various ICU-specific definitions for the first cases of IPA in COVID-19 patients. However, a standard definition for COVID–19–associated pulmonary aspergillosis (CAPA) was proposed (6, 13). 

IPA can be diagnosed using galactomannan (GM) testing. This test detects the presence of galactomannan, a cell wall component of *Aspergillus* species, in serum or bronchoalveolar lavage (BAL) fluid. Studies show that elevated levels of GM, alongside other clinical findings, can indicate IPA in immunocompromised patients (14). However, the use of GM testing for diagnosing CAPA remains a matter of debate as false-positive results may occur due to the cross-reactivity with other fungal infections or non-infectious conditions (15). On the other hand, the clinical features and radiological findings of CAPA are similar to severe COVID-19; therefore, radiological evidence is insufficient to diagnose CAPA (16). Similar tests, such as nasal lavage GM, have been proposed as less invasive methods. However, validation is required in patients with histologically confirmed CAPA (17). 

Although probable/proven CAPA definitions are available, distinguishing between colonization and invasive fungal infection remains challenging (18). This distinction is usually impossible without histopathological evidence or a positive serum GM test (19). This review attempted to gather data on serum and BAL GM levels to determine the value of these tests for CAPA prediction and establishing a diagnostic threshold.

## Materials and methods

### Search Strategy

Articles published by the end of November 2023 in four main databases (ScienceDirect, MEDLINE, Cochrane Library, and PubMed) were assessed comprehensively according to the following search protocol: (aspergillus) OR (aspergillosis) AND (SARS-CoV-2) OR (COVID) OR (2019 nCoV) OR (novel coronavirus) OR (COVID-19) AND galactomannan AND (CAPA). 

### Eligibility criteria

**Fig. 1:**
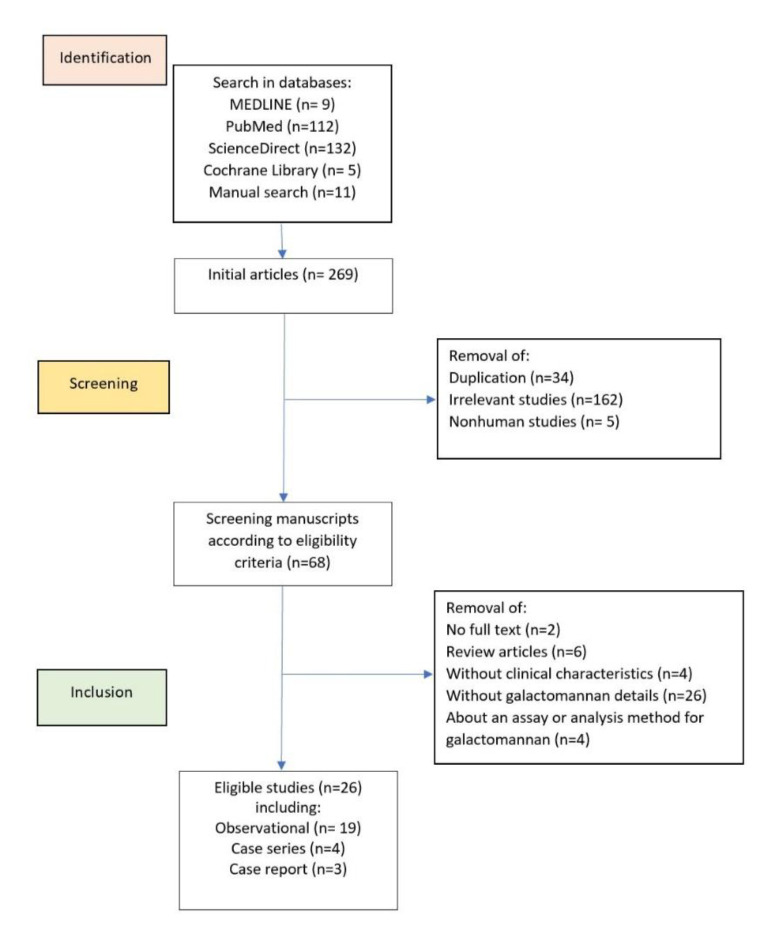
Flowchart of study selection

All human studies in the English language, including case reports and original studies, reporting the level of GM in serum or BAL of patients with COVID-19 diagnosed with possible, probable, or proven aspergillosis, based on the 2020 European Confederation of Medical Mycology (ECMM) and the International Society of Human and Animal Mycology (ISHAM) consensus criteria, were identified [10]. Commentaries, systematic reviews, studies reporting GM as the mean or median, and those that did not report risk factors were excluded. Also, studies conducted to establish or validate a laboratory method for GM were excluded. The flowchart of the study selection is presented in Fig. 1.

ECMM/ISHAM defines three CAPA categories: possible (fungal elements in samples or positive lavage culture), probable (microscopic detection in BAL and mold presence), and proven. Patients in this study were grouped as non-confirmed (possible/probable) or proven CAPA, with GM levels and other factors compared for diagnostic insights.

### Data extraction

A checklist was prepared containing data on study locations and patients’ details, such as age, sex, serum GM level, BAL GM level, the result of PCR or culture and histopathology, *Aspergillus* species, radiological findings, and mortality. The completed checklist is presented in supplementary Index 1. Two internal reviewers assessed the eligibility of the studies and extracted the relevant data. A third reviewer attributed cases of inconsistencies.

### Statistical analysis

To analyze the data, we used SPSS software version 29. First, we used the Shapiro-Wilk test to check the normal distribution of the quantitative variables. We used the independent Student’s T-test if the data were normally distributed to compare the mean variables between the two groups. Otherwise, we used the non-parametric Mann-Whitney test. We used the Chi-square and Fisher's exact test according to the expected values to check the relationship between the qualitative variables. Finally, we used the receiver operating characteristic (ROC) curve analysis to check and visualize the performance of the Two-group classification.

## Results

Initially, 269 articles were identified through database searching. A total of 26 articles were retrieved according to the selection flowchart (20-47). Within these articles, a number of 239 patients were in accordance with the protocol to be used for the predictivity of GM. The median age of the patients was 61 years old (IQR: 53–71), and 171 patients (69.2%) were male. According to the definition, 123 patients (49.8%) were probable, and 124 (50.2%) were proven. The baseline information of the patients, including comorbidities, medication, and radiological findings are presented in Table 1.

**Fig. 2:**
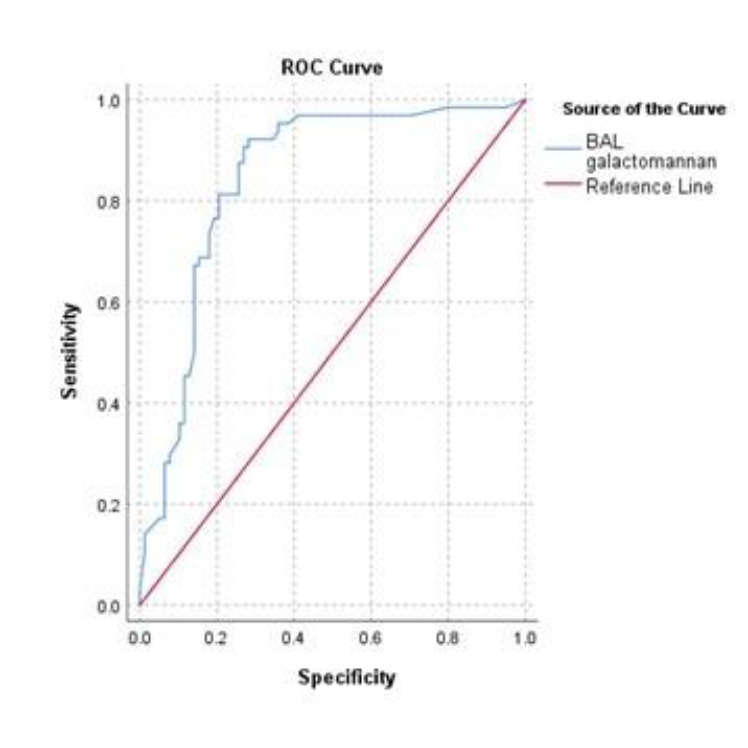
Results of ROC curve for determining BAL GM cut-off

The median of serum GM was 0.51 (0.10, 1.54) in the non-confirmed group and 0.47 (0.1, 1.51) in the proven group (p = 0.73). The median of BAL GM was 0.10 (0.05, 0.89) in the non-confirmed and 2.80 (1.40, 6.00) in the proven group, which was significantly different (p ˂ 0.001). The Area Under Curve (AUC) for serum GM was 0.51, indicating that the test has a 50% chance of distinguishing between confirmed and non-confirmed cases. In other words, the serum GM test has no significant discrimination capacity to distinguish CAPA (Figure 2).

**Table 1:** Baseline information of the patients included in the analysis

Conditions	Number of cases*	Percentage (%)
Comorbidities		
HIV	0	0
Chemotherapy	6	2.4
Cancer	17	6.9
Wegner	1	0.4
Transplant	27	10.9
Stroke	3	1.2
Smoking	9	3.6
SLE	1	0.4
Myasthenia gravis	1	0.4
Obesity	21	8.5
HTN	62	25.1
DM	66	26.7
COPD	24	9.7
Coronary artery disease	13	5.3
Chronic renal failure	7	2.8
HLP	12	4.9
Medication for the treatment of covid-19		
Corticosteroid	109	44.1
Remdesivir	24	9.7
Tocilizumab	28	11.3
Anakinra	1	0.4
Antibiotics		
Meropenem	9	3.6
Imipenem	0	0.0
Levofloxacin	3	1.2
Azithromycin	21	8.5
Linezolid	6	2.4
Piperacillin-Tazobactam	8	3.2
Vancomycin	4	1.6
Radiographic findings		
Consolidation	69	27.9
Hallo sign	69	27.9
Cavitary lesion	35	14.2
Cresent sign	1	0.4
Pleural effusion	13	5.3
Nodules	16	6.5
Pneumothorax	2	0.8

* The data are based on the “reported” information from the articles; missing data should be considered.

With 81.3 % sensitivity and 79.5% specificity, BAL GM cut-off was 1.01 ODI (Optical Density Index). AUC for BAL GM was 0.84, suggesting that the test has a 84% chance of distinguishing between confirmed and non-confirmed groups.

The *Aspergillus *species, which were isolated using Polymerase Chain Reaction (PCR) or culture, were as follows: *Aspergillus fumigatus* in 84 cases (34.0%), *Aspergillus flavus* in 19 cases (7.7%), *Aspergillus niger* in 14 cases (5.7%), *Aspergillus nidulans* in 5 cases (2.0%), and *Aspergillus tammarii* in 1 case (0.4%). 

Subpopulation analysis was performed to evaluate the difference in the median of serum or BAL GM in patients with comorbidities or specific conditions. In patients with hypertension, the median of serum GM in the non-confirmed and proven groups was 0.31 and 0.56, respectively, which is not significantly different. However, in the same population, the median of BAL GM was 0.18 in the non-confirmed group and 3.5 in the proven group (p< 0.001). In patients with diabetes, the median of serum GM was 0.26 in the non-confirmed group and 0.82 in the proven group (p= 0.03). The median of BAL GM in the aforementioned population was 0.16 in the non-confirmed group and 1.65 in the proven group, which was statistically different (p=0.004). This finding may indicate that serum GM may be valuable in some populations, like patients with diabetes mellitus. The details of the assessment of subpopulations are presented in Appendix 1. 

The patients receiving antiviral for COVID-19 were compared. In patients receiving remdesivir, both serum and BAL GM were statistically higher in the proven group (p< 0.05). 

Considering the *Aspergillus* species, the proven and non-confirmed groups exhibited no significant difference, except for *A. fumigatus*. The median level of BAL GM in patients with culture or PCR of *A. fumigatus* was 0.14 and 2.79 in the non-confirmed and proven group, respectively (p ˂ 0.001). Finally, in the patients who died, both levels of serum and BAL GM was significantly higher in proven cases. The details of analysis of populations accompanying some clinical conditions with levels of serum or BAL GM are presented in Appendix 1. 

The median time of diagnosis of aspergillosis after COVID-19 diagnosis was 30 days (IQR: 27.00, 0.54 days) in the non-confirmed group vs. 21 days (IQR: 19.00, 27.00 days) in the proven group (p= 0.002). However, the time between diagnosis of aspergillosis to ICU admission was not significantly different between the two groups (16 days in the non-confirmed and 12.5 days in the proven group, p=0.063). Surprisingly, the death rate in the proven group was significantly lower than in the non-confirmed group (27.6% vs. 72.4%, p< 0.001).

## Discussion

This review collected data on GM results from different studies to propose a predictive level of GM in CAPA patients. According to the present study, the BAL GM level of 1.01 can be used to predict proven CAPA. 

Our study found no diagnostic value for serum GM. This finding aligns with the explanation that due to the clearance of GM from the systemic circulation by neutrophils, the serum GM test may not have the necessary sensitivity to diagnose aspergillosis in non-neutropenic patients (48). Patients with COVID-19 do not have serious neutropenia, so the serum GM test may not be valuable in CAPA diagnosis. In some studies, the positivity rate of serum GM has been reported as low as 20% in CAPA patients (7). However, our results showed that serum GM still applies to some subpopulations, i.e., patients with diabetes mellitus. This finding should be validated in future studies. Moreover, serum GM can be associated with false-positive results in some patients, such as those undergoing amoxicillin-clavulanate or piperacillin-tazobactam treatment (49). In general, various factors such as age, type of underlying immunodeficiency, bacterial infections, nutritional support, and even food consumed throughout the day may cause changes in the sensitivity and specificity of this test (50). Considering this low sensitivity of the serum GM test in CAPA diagnosis and the safety concerns regarding performing diagnostic bronchoscopy, obtaining evidence of invasive aspergillosis in the airways in patients with COVID-19 is challenging (51). This obstacle can be overcome by examining the level of GM in BAL fluid, as the GM antigen is released during the active growth of the fungus in the bronchi (52). The BAL GM level of ≥1.0 is considered positive in diagnosing IPA in neutropenic patients with cancer according to the European Organization for Research and Treatment of Cancer and the Mycoses Study Group Education and Research Consortium) EORTC and MSGERC( (9). A threshold of 0.5–1.5 for BAL GM for diagnosing IAPA and CAPA was established by ECMM/ISHAM based on expert opinions and studies on patients with hematologic malignancies, who are highly prone to aspergillosis (53). The predictive level of 1.01 for diagnosing CAPA presented in our study aligns closely with the level provided in the ECMM/ISHAM consensus criteria (7, 13). Although the sensitivity of BAL GM testing has been reported to be higher than serum GM testing, a combination of clinical, radiological, and laboratory findings is usually recommended for an accurate diagnosis of CAPA (7, 13, 52, 54)

This study is the first review analyzing the data on serum and BAL GM to suggest a predictive level of GM for confirmed CAPA cases. However, it has some limitations. First, the data were extracted from various countries, each with different levels of accuracy in reporting laboratory data, particularly GM. Second, some crucial information was overlooked in the initial data collection. Specifically, details related to the antibiotics, antifungals, and other medications administered for patients with CAPA were not recorded.

## Conclusions

The literature review of the present study analyzed data from patients with CAPA and indicated that serum GM may not be effective in diagnosing CAPA. However, the BAL GM level ≥ 1.01 can be used as a strong predictor of CAPA with 81.3 % sensitivity and 79.5% specificity. This level can help diagnose CAPA, initiate antifungal treatment early, and prevent late-treatment consequences. Nevertheless, further examination of this level in future clinical trials is needed. 

## Figures and Tables

**Table 1:** Summary of the studies on the Value of Galactomannan Test in Diagnosing COVID-19–Associated Pulmonary Aspergillosis

No.	First Author	Year	Type of study	No of cases used in review	Non-confirmed or proven case	PMH	Corticosteroid	Tocilizumab	Remdesivir	Isolated aspergillus species	Death	Reference
1	Mira yulianti	2022	Case series	2	Non-confirmed	Dm, HTN	Yes	Yes: 1	Yes	-	No	
2	Kuo-Lun Wu	2022	Retrospective observational	6	Non-confirmed	Wegner, HTN, DM COPD	Yes	Yes	Yes:2	_	Yes: 2	
3	Dependra Kumar Rai	2022	Retrospective	5	Non-confirmed	DM	No	Yes: 2	2	-	No	
4	Carla M Roman-Montes	2022	Retrospective	2	Non-confirmed		Yes	No	Yes:1	_	Yes: 1	
5	Maria Fernando Benedetti	2021	Case series	2	Non-confirmed	Transplant, DM	No	No	No	_	Yes: 1	
6	Paul Chauvet	2020	Retrospective	1	Non-confirmed	_	Yes	No	No	_	yes	
7	David Ranhell	2021	Case series	5	Non-confirmed	_	No	No	Yes:3	_	No	
8	D Armestrang james	2020	Observational	32	Non-confirmed:19 Proven: 13	Transplant, Cancer, COPD	Yes: 5	No	Yes:2	-	Yes: 8	
9	Jin Lee Lim	2022	Case report	2	Non-confirmed	Transplant	Yes: 1	No	No	_	Yes: 1	
10	Kaiqing leen	2022	Case report	1	Non-confirmed	Transplant	Yes	Yes	Yes	-	No	
11	Sean X. Zhang	2021	Observational	33	Proven	Transplant, Obesity, HTN, DM, Stroke, IHD, COPD	Yes: 12	Yes: 7	-	A. flavus: A. parasiticus A. niger A. fumigatus	Yes: 10	
12	Khaled Alobaid	2021	Case report	1	Proven	Transplant	No	Yes	No	A. niger	No	
13	Andrew M Borman	2022	Retrospective observational	58	Proven: 10 Non-confirmed: 48	_	_	_	_	A. fumigatus	_	
14	Marina macado	2021	Observational	8	Proven	Obesity, HTN, DM, Asthma, Cancer	-	-	Yes:1	A. fumigatus	Yes: 8	
15	AC Martin	2021	Case series	3	Proven	HTN, DM, Obesity	Yes	Yes: 1	_	A. fumigatus	Yes: 1	
16	Zia Hashim	2021	Observational	4	Proven: 3 Non-confirmed: 1	SLE, Obesity, HTN, Transplant	Yes: 4	-	Yes:1	A. fumigatus A. niger	Yes: 3	
17	Hernando Trujillo	2021	Observational	3	Proven	Transplant	No	No	No	A. fumigatus	Yes: 1	
18	Hayato Mitaka	2020	Observational	1	Proven	COPD	Yes	Yes	No	A. fumigatus	Yes	
19	Elizabeth Paramythiotou	2021	Observational	6	Proven	Transplant, Stroke, HTN, DM, COPD, CKD	Yes	No	Yes:2	A. niger A. terreus	Yes: 4	
20	Nosheen Nasir	2020	Observational	6	Proven	DM, HTN,	Yes: 3	Yes: 5	_	A. flavus A. fumigatus A. niger	Yes: 4	
21	Joao N de Almeida	2022	Retrospective observational	14	Proven: 8 Non-confirmed: 6	Cancer, Obesity, HTN, Dm, IHD, COPD, Myasthenia gravis	_	_	_	A. fumigatus A. niger	Yes: 10	
22	Wiiliam Hurt	2021	Observational	18	Non-confirmed	DM, HTN, Obesity, Stroke	Yes: 7	No	No	A. fumigatus A. niger A. flavus	Yes: 9	
23	Masashi Ogawa	2023	Observational	11	Non-confirmed	Cancer, Transplant, HTN, DM	Yes: 11	Yes: 2	-	A. fumigatus A. niger A. terreus	Yes: 7	
24	Luc Desmedt	2024	Retrospective observational cohort	8	Proven:	Transplant, DM, Obesity	Yes: 7	No	-	A. flavus	Yes: 7	
25	Ali Nuh	2023	Retrospective	9	Proven	COPD, HTN, DM, Obesity	-	-	-	A. flavus A. fumigatus	Yes: 3	
26	Tiago Alexandre Cocio	2023	Retrospective	15	Proven	IHD, DM, Stroke, HTN, Hypothyroidism, Tuberculosis, Obesity, Parkinson	Yes: 15	-	-	A. fumigatus A. flavus A. tamari A. niger A. terreus	Yes: 10	

Abbreviations: CKD: Chronic Kidney Disease, COPD: Chronic Obstructive Pulmonary Diseaes, DM: Diabetes Mellitus, HTN: Hypertension, IHD: Ischemic Heart Disease, SLE: Systemic Lupus Erythematous.
